# Niemeyer Virus: A New Mimivirus Group A Isolate Harboring a Set of Duplicated Aminoacyl-tRNA Synthetase Genes

**DOI:** 10.3389/fmicb.2015.01256

**Published:** 2015-11-10

**Authors:** Paulo V. M. Boratto, Thalita S. Arantes, Lorena C. F. Silva, Felipe L. Assis, Erna G. Kroon, Bernard La Scola, Jônatas S. Abrahão

**Affiliations:** ^1^Laboratório de Vírus, Departamento de Microbiologia, Instituto de Ciências Biológicas, Universidade Federal de Minas GeraisBelo Horizonte, Brazil; ^2^URMITE CNRS UMR 6236 – IRD 3R198, Aix Marseille UniversitéMarseille, France

**Keywords:** *Mimiviridae*, Niemeyer virus, aminoacyl-tRNA synthetase, gene duplication, giant virus isolation

## Abstract

It is well recognized that gene duplication/acquisition is a key factor for molecular evolution, being directly related to the emergence of new genetic variants. The importance of such phenomena can also be expanded to the viral world, with impacts on viral fitness and environmental adaptations. In this work we describe the isolation and characterization of Niemeyer virus, a new mimivirus isolate obtained from water samples of an urban lake in Brazil. Genomic data showed that Niemeyer harbors duplicated copies of three of its four aminoacyl-tRNA synthetase genes (cysteinyl, methionyl, and tyrosyl RS). Gene expression analysis showed that such duplications allowed significantly increased expression of methionyl and tyrosyl aaRS mRNA by Niemeyer in comparison to APMV. Remarkably, phylogenetic data revealed that Niemeyer duplicated gene pairs are different, each one clustering with a different group of mimivirus strains. Taken together, our results raise new questions about the origins and selective pressures involving events of aaRS gain and loss among mimiviruses.

## Introduction

Since the discovery of the first member of the family *Mimiviridae*, *Acanthamoeba polyphaga mimivirus* (APMV), in 2003, more mimivirus-like viruses are being isolated with increasing frequency from phagotrophic protists (La Scola et al., 2003). Mimivirus-like particles have been detected in the most diverse environments, such as rivers, soil, oceans, hospital and animals, and from different countries, such as France, Tunisia, Chile, Australia among others (La Scola et al., 2010; Arslan et al., 2011; Boughalmi et al., 2013). Recently, Campos et al. (2014) described the discovery of the first giant virus isolated in Brazil, named *Samba virus* (SMBV), which was isolated in 2011 from surface water collected from the Negro River, in the Amazon forest (Campos et al., 2014). SMBV is biologically and molecularly related to other mimiviruses, and was isolated in association with Rio Negro virus (RNV), a novel virophage strain belonging to this new class of viruses that parasitize the viral factory during mimivirus replication (Campos et al., 2014). Currently, the family *Mimiviridae* consists of dozens of mimivirus-like isolates that are able to infect amoeba of the genus *Acanthamoeba.* These viruses have been grouped into three distinct lineages, according to their polymerase B gene sequence and other genetic markers: lineage A (containing APMV), lineage B (containing *Acanthamoeba polyphaga moumouvirus*) and lineage C (containing *Megavirus chilensis*) (Gaia et al., 2013). These amoeba-associated viruses have led to a paradigm shift in virus research due to their peculiar features which had never been seen in other viruses until then: large viral particles presenting a diameter of approximately 750 nm, covered by capsid associated fibers, and containing large double stranded DNA genomes of about 1.2 megabases (Mb), and approximately 1000 hypothetical proteins, many of them still uncharacterized or having functions never/rarely seen before in other viruses (La Scola et al., 2003; Raoult et al., 2004). Among the most intriguing predicted proteins in the genome of mimiviruses, it is worth highlighting those related to DNA repair and translation machinery, as well as chaperones related to DNA processing (La Scola et al., 2003; Raoult et al., 2004). Genes that encode translation related proteins, such as aminoacyl tRNA synthetases (aaRS) and translation factors, could hypothetically confer on APMV and other giant viruses a certain degree of autonomy from cellular machinery, and may be under conservative selection pressure (Raoult et al., 2004). Currently, seven aaRS have already been described among mimiviruses genomes: tyrosyl, cysteinyl, methionyl, arginyl, isoleucyl, asparaginyl, and tryptophanyl tRNA-synthetases. Among the aforementioned molecules, the first four enzymes are encoded by the genome of APMV, the prototype of the family *Mimiviridae.* However, no aaRS duplication events in the family *Mimiviridae* have been previously reported, other than in the exceptional case of the *Acanthamoeba polyphaga moumouvirus*, that possesses four orthologs of arginyl-tRNA synthetase in its genome (Yoosuf et al., 2012). In this work we describe the isolation and characterization of Niemeyer mimivirus, a new mimivirus-like virus isolated from water samples from an urban lake in Brazil. Genomic data show that Niemeyer harbors duplicated copies of three of its four aaRS genes (cysteinyl, methionyl, and tyrosyl aaRS), which are associated with increased expression of methionyl and tyrosyl aaRS mRNA by this virus in comparison to APMV. Remarkably, phylogenetic data revealed that Niemeyer duplicated genes are different from each other, each one clustering with a different group of mimivirus strains. Taken together, our results raise new questions about the origins and selective pressures involving aaRS gain and loss events among mimiviruses.

## Materials and Methods

### Sample Collection and Virus Isolation

**FIGURE 1:**
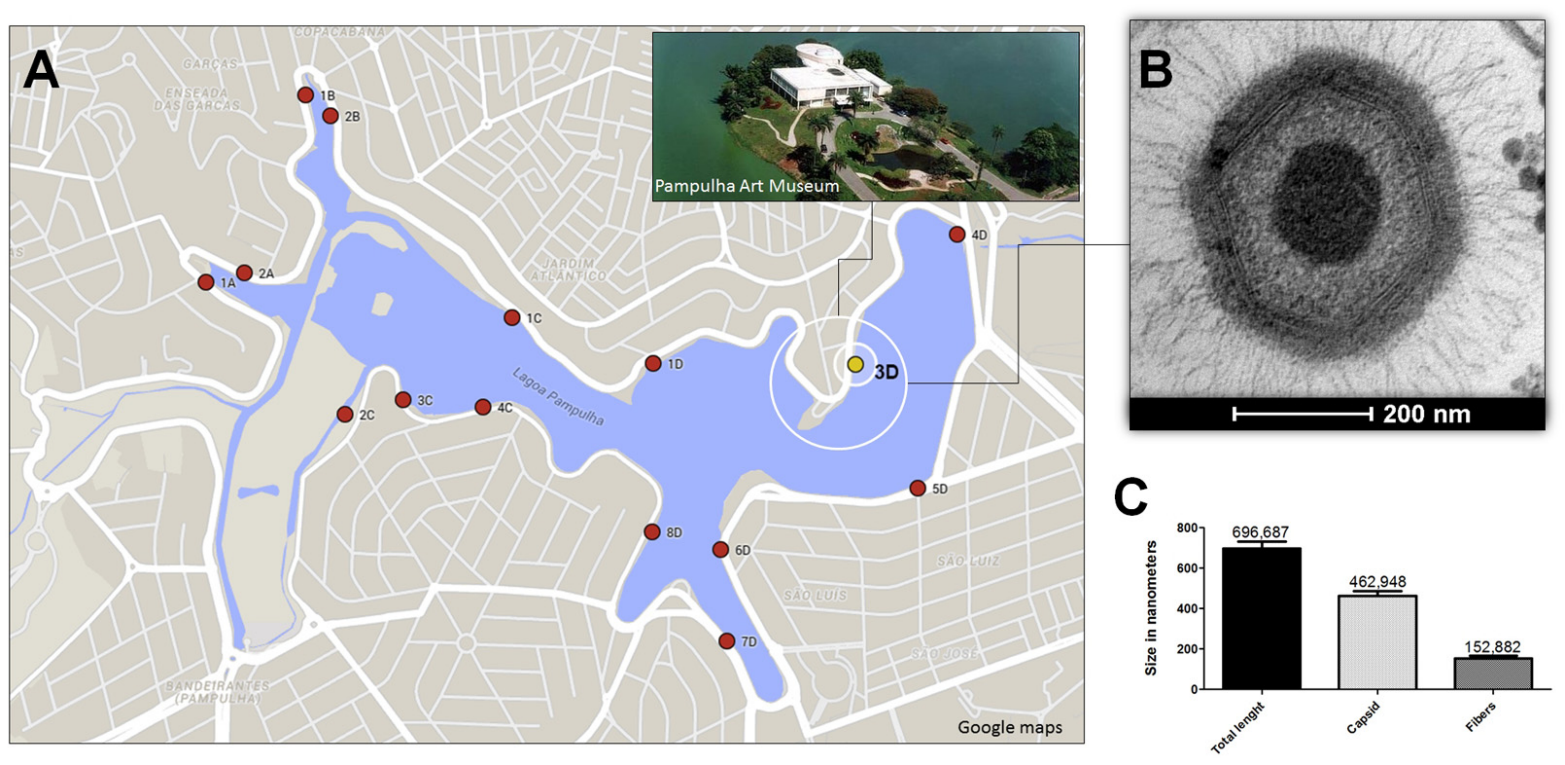
**Niemeyer virus (NYMV) collection site, morphometric analysis, and electron microscopy.** **(A)** Shows a map of the Pampulha Lagoon, from where the samples were collected. Red dots: collection points; yellow dot: NYMV isolation site. **(B)** Shows an image of a NYMV particle, observed in electron microscopy. **(C)** Shows the size, in nanometers, of different components of the NYMV particle: only the fibers, only the capsid and the particle as a whole.

To explore the presence of giant viruses in an urban lake marked by a high concentration of organic matter, in 2011 we collected about 80 water samples, located at equidistant points, around Pampulha Lagoon (19°51′0.60″S and 43°58′18.90″W), in the city of Belo Horizonte, Brazil (**Figure 1A**). After collection, the samples were stored at 4°C overnight. Then, 500 μl of each sample was added to 4.5 mL of autoclaved rice and water medium made with 40 rice grains in 1 l of water. The samples were stored for 20 days in the dark at room temperature. Afterward, 5 × 10^3^ *Acanthamoeba castellanii* trophozoites (ATCC 30234), kindly provided by the Laboratório de Amebíases (Departamento de Parasitologia, ICB/UFMG) were added, and the samples were re-incubated under the same conditions for 10 days (Dornas et al., 2014). After the enrichment process, samples were pooled in groups of five, and filtered through a 1.2 μm membrane to remove impurities, and a 0.2 μm membrane to retain giant viruses. The samples were then subjected in parallel to real-time PCR, targeting the RNA helicase gene (primers: 5′ACCTGATCCACATCCCATAACTAAA3′ and 5′GGCCTCATCAACAAATGGTTTCT3′) and to viral isolation from *A. castellanii*. As a control for the molecular and biological assays APMV was used. A new virus isolate, Niemeyer mimivirus, was grown and purified as described by La Scola et al. (2003) and Abrahão et al. (2014), respectively. The virus was called Niemeyer in tribute to the Brazilian architect who designed important buildings all over the world, including the Pampulha Art Museum near to where the virus was isolated (**Figure 1A**).

### NYMV Virus Transmission Electron Microscopy

For the electron microscopy assays, *A. castellanii* cells were cultivated until 80–90% confluence was observed and infected with NYMV in a M.O.I of 0.01. Twelve hours post-infection (hpi), when approximately 50% of the trophozoites were presenting cytopathic effects, the medium was discarded and the monolayer gently washed twice with 0.1 M sodium phosphate buffer. Samples were fixed by adding glutaraldehyde (2.5% v/v) for 1 h at room temperature. The cells were then collected by centrifugation at 1500 *g* for 10 min, the medium was discarded and the cells were stored at 4° C until electron microscopy analysis was performed.

### Evaluation of the Replication Profile of NYMV

Briefly, NYMV was inoculated in *A. castellanii* cells until appearance of cytopathic effect and purified by centrifugation on a 25% sucrose cushion as previously described (Abrahão et al., 2014). The titer was obtained by using the Reed–Muench method. To evaluate the replication profile of NYMV, the procedure was performed in 96-well Costar^®^ microplates (Corning, NY, USA) containing 40,000 cells of *A. castellanii* maintained in 100 μl of PAS (Page’s amoeba saline, PAS) culture medium per well. The cells were then infected with NYMV at a multiplicity of infection (M.O.I.) of 10. The cells were collected at different time points (0, 1, 2, 4, 8, and 24 hpi) and submitted to cell counting with a Neubauer chamber to evaluate the reduction of cells and the cytopathic effect. As a control for this experiment we used APMV, which was kept under the same conditions as NYMV.

### Genome Sequencing and Annotation

The genome of NYMV was sequenced using the Illumina MiSeq instrument (Illumina Inc., San Diego, CA, USA) with the paired-end application. The sequenced reads were imported to CLC_Bio software^1^ and assembled into contigs by the *de novo* method. The prediction of open reading frame (ORF) sequences was carried out using the FgenesV tool. ORFs smaller than 100aa were excluded from the annotation. Paralogous groups of genes were predicted by OrthoMCL program. The ORFs were functionally annotated using similarity analyses with sequences in the NCBI database using BLAST tools. In addition, the presence of trademark genes of the family *Mimiviridae* was evaluated, and some of them were analyzed in detail. Genbank number: KT599914.

### Similarity Analysis

Viruses of the genus *Mimivirus* are divided into groups A to C. Thereby, the ORFs predicted in NYMV genome were compared to amino acid sequences available in Genebank of APMV (group A), APMOUV (Group B), and MCHV (group C), as well as sequences from SMBV (group A), a Brazilian mimivirus isolate. The AAI calculator program^2^ Rodriguez-R and Konstantinos (2014) was used to estimate the average amino acid identity between two protein datasets using both best hits (one-way AAI) and reciprocal best hits (two-way AAI). The similarity and score thresholds for the alignments were 70 and 0%, respectively. A minimum alignment of 50% was considered.

### Expression of Translation-related Genes

In order to check the expression of aaRS by NYMV, we selected four genes based on the APMV genome sequence (methionyl, tyrosyl, cysteinyl, and arginyl tRNA synthetases) to evaluate the expression profile of NYMV in comparison with APMV as previously described by Silva et al. (2015). Twenty-four-well plates containing 1 × 10^5^ amoeba per well, kept in PAS medium were infected with NYMV and APMV at M.O.I. 10 and incubated at 32°C for 8 h. Cells were collected, centrifuged and the pellet used for total RNA extraction, reverse transcription and quantitative PCR. Briefly, total RNA was extracted using the RNeasy kit (Qiagen, Germany), and reverse transcription was performed by using the MMLV reverse transcriptase (Promega, USA), as recommended by the manufacturers. The cDNA was used to determine the levels of aaRS mRNA by quantitative PCR (primers in **Table 1**) by using specific primers, SYBR Green Master Mix (Applied Biosystem, USA) and water in 10 μL reactions. Reactions were carried out in a StepOne instrument (Applied Biosystem, USA). All reactions had been previously optimized and presented high efficiency values. Relative gene expression analyses were performed using the ΔΔCt method and normalized to the expression of 18S ribosomal RNA (18S rDNA) and the viral RNA helicase mRNA and calibrated using the lower value (=1). Statistical analysis and primers sequences were as described by Silva et al. (2015).

**Table 1:** Primers used for quantitative PCR.

Gene	Foward primer	Reverse primer
Leucyl-tRNA	GGGATTCGAACCCACGACAT	ATAAGCAAAGGTGGCGGAGT
Histidyl-IRNA	TTAGTGGTAGAACTACTGTTTGTGG	TTTTCAAAAATGACCCGTACAGGAA
Cysteinyl-tRNA	ACAGTCAAaGGATCGTTAGC	AGGATCGTATCAGAATTGAACTGA
Tryptophanyl-tRNA	GTG CAACAATAG ACCTGTTAGTTTA	ACCGGAATCGAACCAGTATCA
Methionyl tRNA synthetase	TGATTGGCGTGAATGGCTGA	ACCAATCACACTAGCCGGAA
Arginyl tRNA synthetase	GTGGGTGATTGGGGAAaCA	TGATACGGTCTCCAATCGGG
Tyrosyl tRNA synthetase	TTTGGCAAACCAATCGGCAA	TGGTTTTGAACCTAGTGGTCGT
Cysteinyl tRNA synthetase	TGCCAACCAGGTACACCAAA	TGCTCTTTGGAAAGGTCGATCA
18S rDNA	TCCAATTTTCTGCCACCGAA	ATCATTACCCTAGTCCTCGCGC
Viral RNA helicase	ACCTGATCCACATCCCATAAaAAA	GGCCTCATCAACAAATGGTTTCT


### Phylogeny

The β-DNA polymerase sequence of NYMV was aligned with sequences from other giant viruses, previously deposited in GenBank, using the ClustalW program. After the alignment analysis, phylogeny reconstruction was performed using the maximum likelihood method implemented by the MEGA5 software. Additionally, sequences of aaRS predicted in the genome of NYMV were aligned with sequences from other giant viruses among GenBank sequences as described above, and the phylogeny reconstruction was performed using the neighbor-joining method in the MEGA5 software.

## Results

### Niemeyer: A New Mimivirus Group A Isolate

Here we report the isolation of Niemeyer virus (NYMV) from water samples collected in an urban lake in Brazil. The isolation was confirmed by the observation of a cytopathic effect in cells of *A. castellanii* (ATCC 30234) after 4 days of incubation and also by viral RNA helicase gene amplification in qPCR assays, a highly conserved gene amongst mimiviruses.

Electron microscopy and morphometric assays showed virus particles with average size of 616 nm in total, with fibers of about 153 nm and a capsid size of about 463 nm (**Figures 1B,C**), similar to the dimensions described for other mimivirus-like viruses. Large viral factories were observed in the amoebic cytoplasm, and these contained viral particles at distinct steps of morphogenesis. In addition, NYMV demonstrated a similar pattern of replication to APMV in one-step-growth curve assays (**Figure 5A**).

### NYMV Genome Analyses and Phylogeny

The final genome assembly of NYMV yielded 69 contigs, consisting of fifteen small contigs (<2,000 bp) and fifty-four large contigs (>2,000 bp), including five contigs larger than 100 kb. The NYMV genome is a double-stranded DNA molecule composed of approximately 1,299,140 base pairs. This genome presented a mean C–G content of 27.96%, which is similar to that of other mimiviruses. A total of 1003 proteins were predicted, ranging in size from 100 to 2156 amino acids, with a mean size of 379 amino acids. Moreover, we identified 970 proteins with high similarity (coverage > 90%; identity > 80%; *e*-value < 10e-5) to mimivirus sequences available in the non-redundant NCBI protein database, as well as 27 proteins with low similarity (coverage 50%; identity 35–80%; *e*-value < 10e-5) to mimivirus sequences. We identified two proteins with higher similarity to non-viral sequences (ID 80: Ankyrin repeat protein – *Trichomonas vaginalis* [coverage: 83%; identity: 29%; *e*-value: 5e-5]; ID 193: Hypothetical protein – *Volvox carteri* [coverage: 99%; Identity: 36%; *e*-value: 8e-12]). Indeed, four putative proteins of NYMV had no significant hit (*e*-value threshold 1e-2) against the NCBI non-redundant sequence database. A total of 90 clusters consisting of 269 paralogous proteins were identified in the NYMV genome, which is a remarkably higher number than that described for other mimiviruses. It is worth mentioning that neither virophage sequences nor other mobilome elements were detected in the analyzed data set.

A comparative analysis of NYMV gene content with other mimivirus sequences was performed, which showed the highest identity and bit-score distributions against mimivirus group A sequences, such as SMBV (**Supplementary Figure S1A**) and mimivirus (**Supplementary Figure S1B**). Moreover, the similarity decreased toward moumouvirus (**Supplementary Figure S1C**) and *Megavirus chilensis* (**Supplementary Figure S1D**) of groups B and C, respectively. Furthermore, the one-way and two-way best hit analysis (**Table 2**) corroborated the previous observations. This analysis showed a two-way similarity higher than 99% for NYMV with both SMBV and APMV, reinforcing its grouping with other mimiviruses in group A. During functional annotation, we identified important proteins required for virus replication: DNA polymerase, helicases, nucleases, and proteins with DNA polymerase sliding clamp activity related to replication processes; resolvases and topoisomerases related to DNA manipulation and processing; transcription and translation factors; and ATPases for DNA packaging. However, no chaperone molecules were detected in the NYMV genome, as has been described elsewhere (Yutin and Koonin, 2009, Yutin et al., 2009). Furthermore, we identified four regions encoding tRNA molecules for leucine (two sequences), histidine and cysteine amino acids. However, unlike other mimivirus genomes, no tryptophan tRNA gene was detected.

**Table 2:** Best-hit analysis of proteins predicted in NYMV genome.

Compared strains	Best hits	Identity (%)	SD (%)	proteins
NYMV × APMV	One-way AAI 1	98,65	4,35	975
(group A)	One-way AAI 2	99,94	1,01	841
	Two-way AAI	99,99	0,08	836
NYMV × SMBV	One-way AAI 1	98,62	4,42	956
(group A)	One-way AAI 2	99,94	1,02	825
	Two-way AAI	99,99	0,08	820
NYMV × Moumou	One-way AAI 1	74,98	3,62	61
(group B)	One-way AAI 2	75,3	3,85	58
	Two-way AAI	75,21	3,7	53
NYMV × MCHV	One-way AAI 1	75,82	5,3	55
(group C)	One-way AAI 2	ND	ND	ND
	Two-way AAI	ND	ND	ND


*NYMV, Niemeyer virus; APMV, *Acanthamoeba polyphaga mimivirus*; SMBV, Samba virus; MCHV, *Megavirus chilensis;* APMOUV, *Acanthamoeba polyphaga moumouvirus;* SD, standard deviation; AAI, average amino acid identity.*

### NYMV aaRS Analyses

**FIGURE 2:**
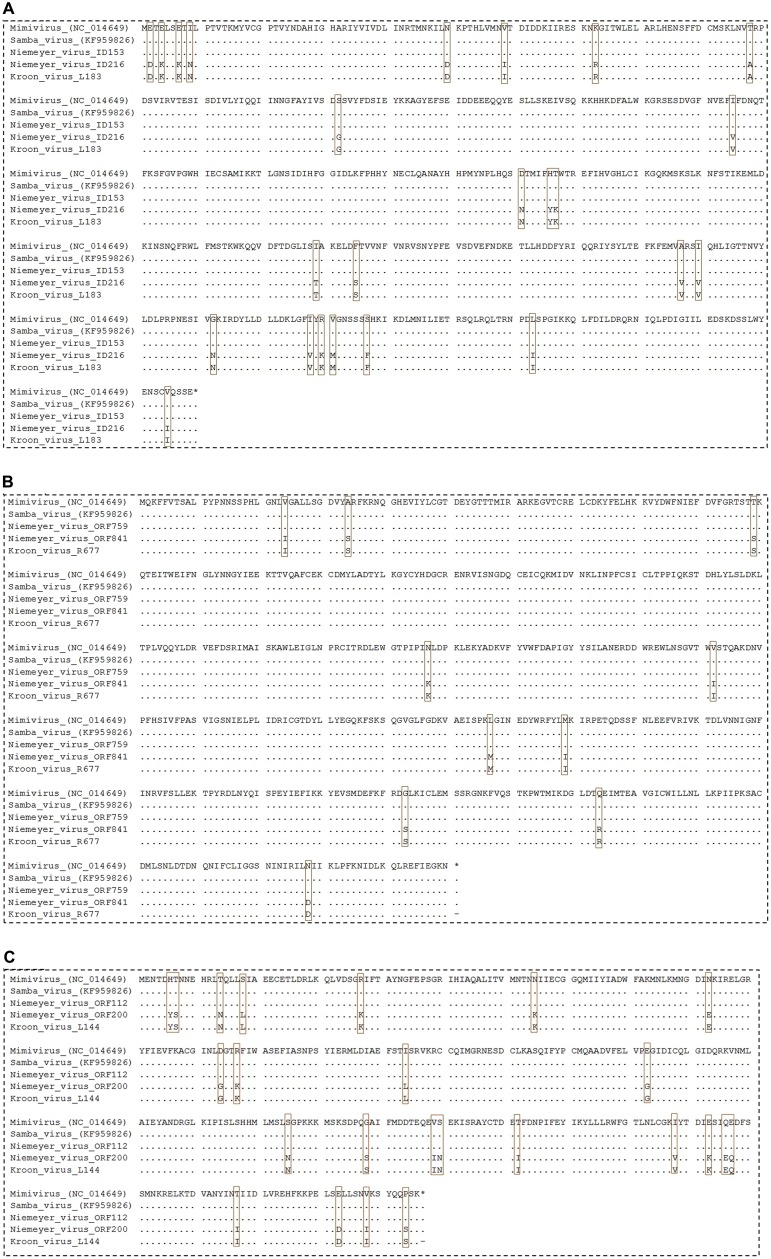
**Alignment of NYMV aaRs sequences.** **(A)** Cysteinyl-tRNA synthetase, **(B)** methionyl-tRNA synthetase, and **(C)** tyrosyl-tRNA synthetase sequences. In this analysis sequences from *Acanthamoeba polyphaga mimivirus* (APMV), the prototype of *Mimivirus*, and sequences from Samba virus (SMBV) and Kroon virus (KROV) were used. Brown boxes highlight polymorphisms in the alignments.

**FIGURE 3:**
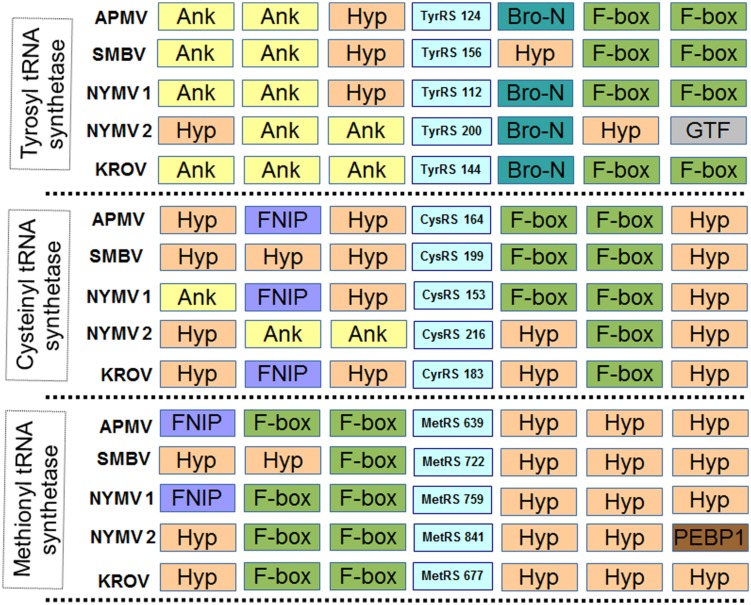
**Neighboring gene synteny analyses of aminoacyl-tRNA synthetase.** Three upstream and downstream neighbor genes of each aaRS predicted in the genome of NYMV and other mimivirus were checked. Ank, ankyrin repeat family protein; Hyp, Hypothetical Protein; F-box, F-box protein family; Bro-n, bro-n family protein; GTF, glycosyltransferase; FNIP, FNIP repeat-containing protein; PEBP1, phosphatidylethanolamine-binding protein. NYMV 1 and 2 refer to each copy of the aaRS genes.

In addition, we evaluated the presence of the landmark aaRS in NYMV genome. The analysis of such proteins is particularly interesting due to the fact that no virus outside the family *Mimiviridae* has been predicted to encode them. We identified seven aaRS sequences in NYMV genome, being two orthologs each for tyrosyl, cysteinyl, and methionyl-tRNA synthetases, as well as one sequence for arginyl-tRNA synthetase. The NYMV genome encoded similar aaRS molecules as those detected in the APMV, Mamavirus, SMBV, and Hirudovirus genomes (mimivirus group A), except for Terra1 virus (mimivirus group C). However, no aaRS duplications were observed in such genomes. The aaRS distribution in other mimivirus genomes, including NYMV, can be seen in **Table 3.** The arginyl-tRNA synthetase encoded by NYMV presented 100% identity (amino acid) with the sequences of Mimivirus and SMBV, whereas the duplicated aaRS of NYMV presented some polymorphisms when paralogs were compared with each other (**Figure 2**). Curiously, one copy of each NYMV duplicated aaRS presented 100% identity with APMV and SMBV, while the other copy had 100% identity with Kroon virus (KROV; **Figure 2**), a Brazilian mimivirus-like virus strain isolated from a water sample collected in an urban lake, at Lagoa Santa city, Brazil (approximately 30 km from Pampulha lagoon). The biological and molecular characterization of KROV is in progress, but preliminary results suggest a possible dichotomy among Brazilian mimivirus A isolates. To evaluate the distribution of duplicated aaRS genes within NYMV and other mimivirus genomes, three upstream and downstream genes neighboring aaRS from APMV, SBMV, KROV, and NYMV were analyzed (**Figure 3**). We observed that the duplicated aaRS in the genome of NYMV are not in tandemly duplicated, being located distant from each other (**Figure 3**). The methionyl-tRNA loci presented the best neighbor gene synteny, as all virus strains had two neighboring genes the same on both sides, with the exception of the SMBV strain, which had a distinct neighbor gene at second position toward the 3′ extremity. Altogether, no conservative genomic loci were observed for any aaRS, and none of the analyzed virus strains shared the same neighboring gene for all of the aaRS analyzed, reinforcing the uniqueness of each isolate (**Figure 3**).

**Table 3:** Distribution of aminoacyl-tRNA synthetases in mimiviral genomes.

Aminoacyl-tRNA sintetase	Mimivirus strains								
	
	NYMV	APMV	Mamavirus	SMBV	Hirudovirus	APMV-M4	MCHV	APMOUV	Terra1 virus
Tyrosyl	2	1	1	1	1	0	1	1	1
Cysteinyl	2	1	1	1	1	1	1	1	1
Methionyl	2	1	1	1	1	1	1	1	1
Argiryl	1	1	1	1	1	1	1	4	1
Isoleucyl	0	0	0	0	0	0	1	1	0
Asparaginyl	0	0	0	0	0	0	1	0	0
Tryptophanyl	0	0	0	0	0	0	1	0	0


*NYMV, Niemeyer virus; APMV, Acanthamoeba polyphaga mimivirus; SMBV, Samba virus; MCHV, Megavirus chilensis; APMOUV, Acanthamoeba polyphaga moumouvirus.*

### Expression of *aaRS* Genes and NYMV Replication Profile

**FIGURE 4:**
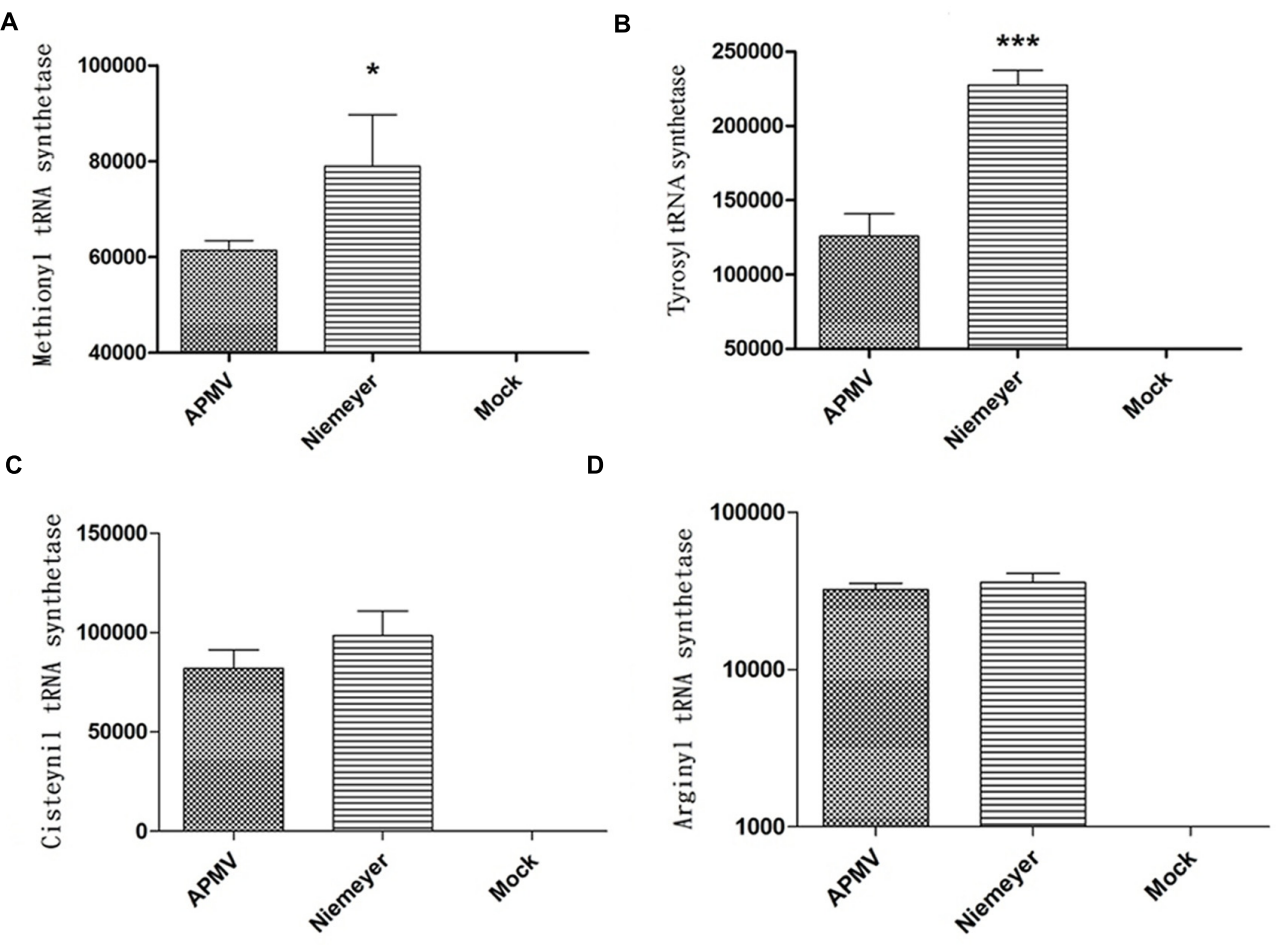
**Aminoacyl-tRNA-synthetase (aaRS) mRNA expression by NYMV.** **(A)** Methionyl tRNA synthetase, **(B)** tyrosyl tRNA synthetase, **(C)** cysteinyl tRNA synthetase, and **(D)** arginyl tRNA synthetase. Relative gene expression analyses were performed using the ΔΔCt method and normalized to the expression of 18S ribosomal RNA [18S rDNA) and the viral RNA helicase mRNA (and calibrated with the lower value (=1)]. The values were subjected in different combinations to one-way ANOVA tests and Bonferroni post-tests (95% confidence intervals). Differences between groups were considered significant when the *p*-values were smaller than 0.05 (asterisks). *Y* axis represents relative abundance.

**FIGURE 5:**
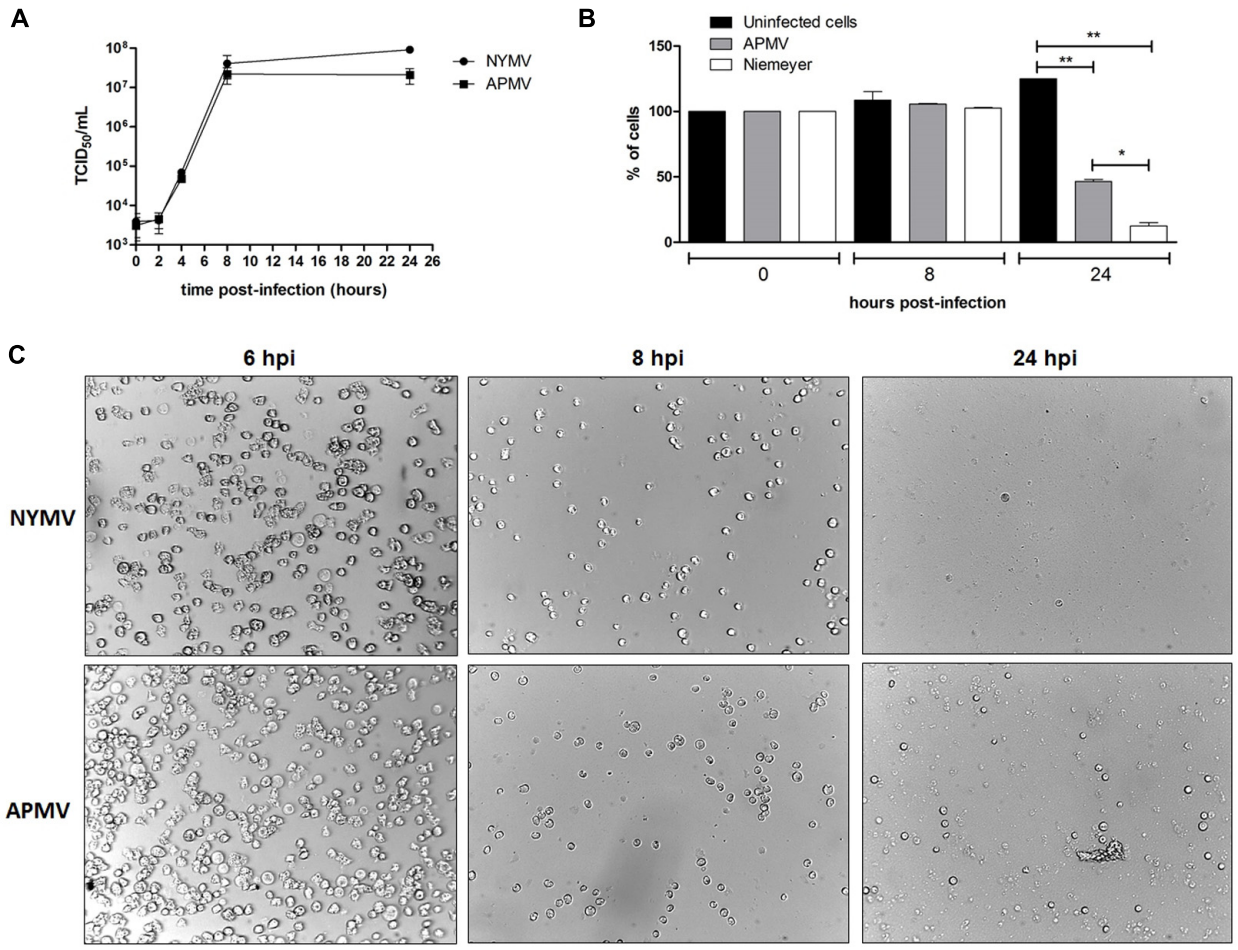
**(A)** One step growth curves of NYMV and APMV in *Acanthamoeba castellanii*. At 6 hpi cells are similar negative control cell (data not shown). **(B)** *A. castellanii* cells counting at different time points (0, 8, and 24 hpi with APMV or NYMV). **(C)** Images obtained from cells of *A. castellanii* infected with NYMV and APMV at different time points after infection.

To evaluate the expression profile of NYMV-encoded aaRS during infection, infected *A. castellanii* cells were collected, processed and assayed by Real-time PCR. The results of quantitative PCR were expressed as arbitrary units, fitted to standard curves generated for each target gene and normalized by amoebal 18S rDNA gene levels. Our results revealed that the expression of two aaRS, methionyl-RS, and tyrosyl-RS (both duplicated in NYMV), was significantly distinct between APMV and NYMV (*p* < 0.001 or *p* < 0.01; **Figures 4A,B**), while for the other two analyzed genes, cysteinyl-RS, and arginyl-RS, there was no significant difference in expression between the two viruses (**Figures 4C,D**). Evaluation of the replication profile of NYMV showed that up to 4 hpi, NYMV and APMV showed a similar replication profile. After 8 hpi it was possible to notice an increased lysis of cells infected with NYMV when compared to APMV. After 24 hpi, the lysis induced by NYMV is greater than that induced in the amoebae infected with APMV (**Figure 5**).

### NYMV Phylogenetic Analysis

**FIGURE 6:**
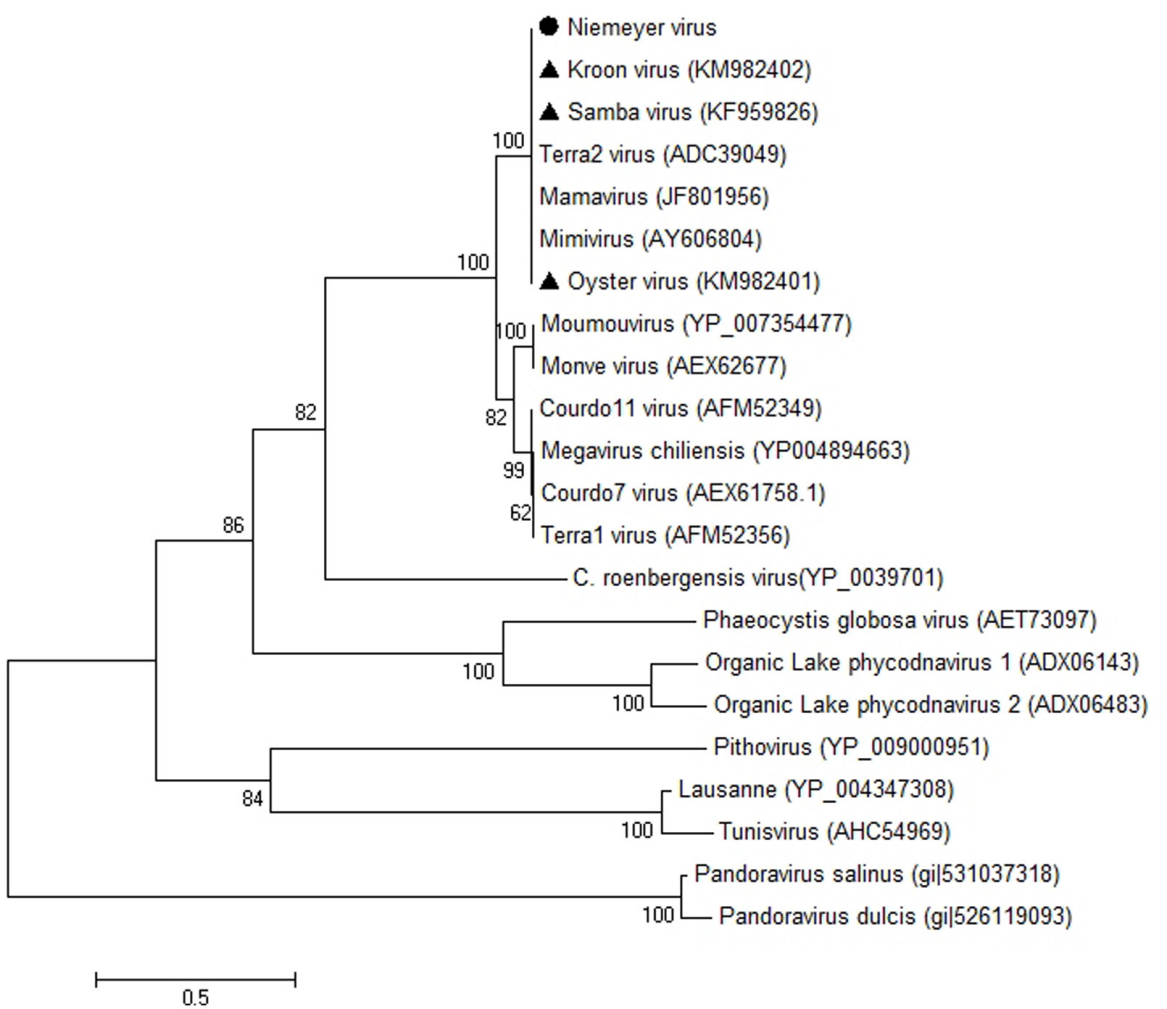
**Phylogenetic reconstruction of mimiviruses including the Brazilian Niemeyer mimivirus strain, based on the Polymerase-B amino acid sequences, using MEGA5 software (Maximum-likelihood – 1,000 bootstrap replicates).** The NYMV (highlighted by black dot) clustered with other Brazilian mimivirus isolates (triangles), as well as other members of mimivirus lineage A. For each sequence, the gene identification number is indicated.

**FIGURE 7:**
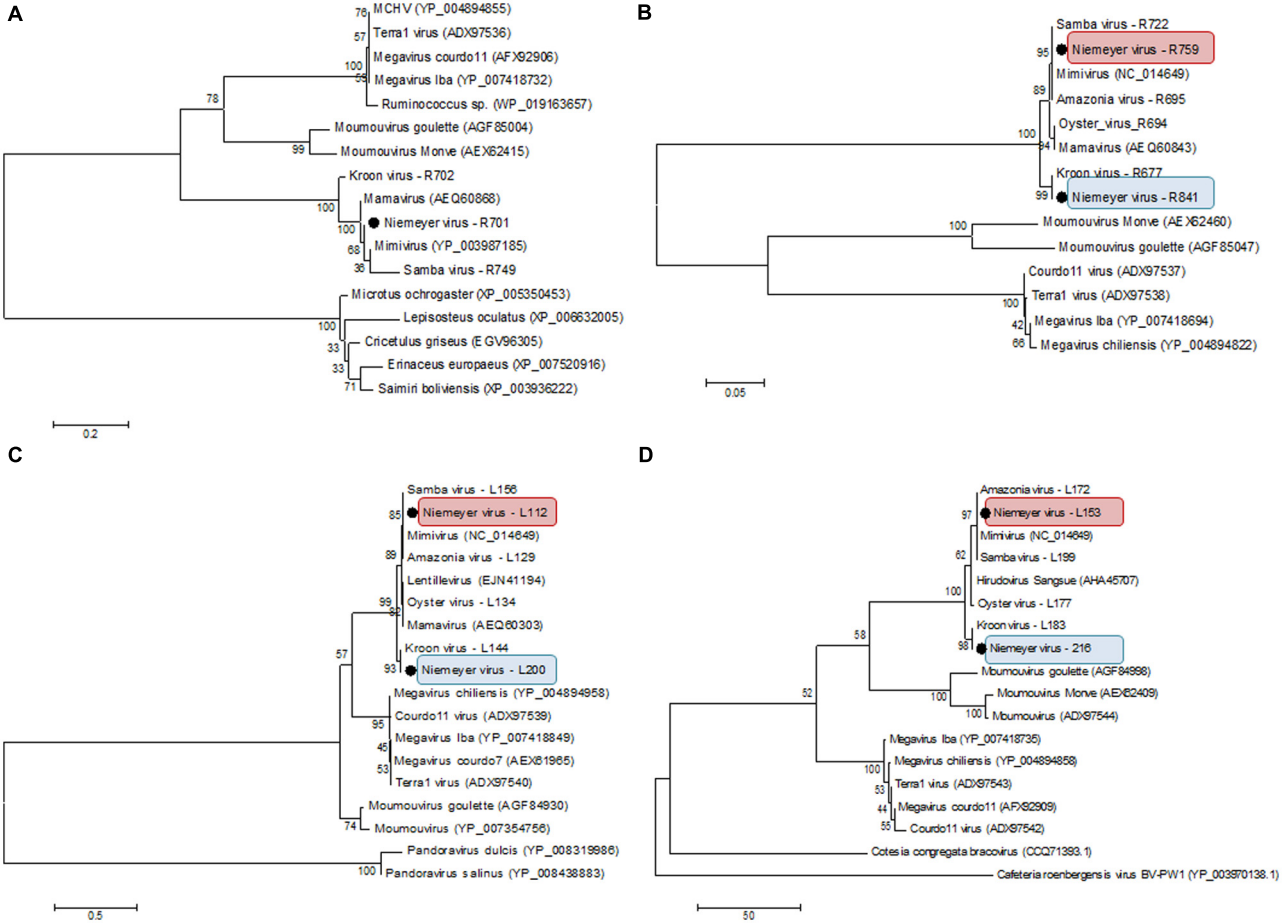
**Phylogenetic analysis based on sequences of **(A)** Arginyl-tRNA synthetase, **(B)** Methionyl-tRNA synthetase, **(C)** Tyrosyl-tRNA synthetase, and **(D)** Cysteinyl-tRNA synthetase predicted in the genome of NYMV, and orthologous sequences obtained from the NCBI database.** The trees were inferred by using MEGA5 software (Neighbor-joining – 1,000 bootstrap replicates). The aaRS encoded by NYMV are highlighted by black dots. For each sequence, the gene identification number is indicated. Red and blue boxes indicates the different copies of NYMV aaRS.

β-DNA polymerase-based phylogenetic analysis corroborated all of the previous observations, clustering the mimivirus NYMV strain with members of *Megavirales* order group A, which includes APMV (the prototype of the family), mamavirus, and other Brazilian mimivirus isolates, such as the SMBV and KROV strains (**Figure 6**). Additionally, we performed aaRS-based phylogenetic analyses of NYMV. The arginyl-tRNA synthetase-based tree (**Figure 7A**) grouped the NYMV within the APMV, SMBV, and Mamavirus branch, with KROV being positioned more distant from the other mimiviruses of group A. The subsequent phylogenetic trees (**Figures 7B–D**), based on duplicated aaRS, presented a peculiar feature, in which one of each doublet grouping within the mimivirus group A branch, and the other copy grouping more distantly with the sequences of the KROV (**Figures 7B–D**).

## Discussion

In this work we describe the isolation of NYMV, a new mimivirus group A isolate from an aquatic habitat marked by a high concentration of organic matter, the eutrophicated urban lake Pampulha Lagoon (**Figure 1A**). NYMV presented some features similar to other mimiviruses such as viral particles of approximately 696 nm and the presence of fibers around the capsid; a replication profile similar to APMV; and gene content/similarity resembling viruses belonging to the genus *Mimivirus*. However, as demonstrated by genomic data and gene expression analysis, NYMV harbors duplicated copies of three of its four aaRS genes, which may be associated with an increased expression of methionyl and tyrosyl aaRS mRNA.

Gene duplication is an important process driving molecular evolution, allowing the formation of new genes with new or redundant biological functions, affecting the evolutionary history and/or the fitness of the organisms. This process is well described for several species in the different domains of life, especially for the eukaryotes (Zhang, 2003). Simon-Loriere et al. (2013) showed, by using comparative analysis among 55 species of RNA viruses from humans, animals, and plants, distributed across 19 viral families and 30 genera, that genetic duplication seems to have only a modest role in the evolutionary history of RNA viruses (Simon-Loriere et al., 2013). Nonetheless, this process has been described quite commonly for DNA viruses (Shackelton and Holmes, 2004). In viruses belonging to the family *Mimiviridae*, gene duplication events are an open field for new studies. It has already been shown that these processes were very important in shaping the APMV genome during its evolutionary history, with about of one-third of the viral genes having at least one other related gene in the same genome (Suhre, 2005).

Despite this, the duplication of aaRS genes related to protein translation, seems not to be a very frequent event in giant viruses, as demonstrated in **Table 3**, in which only two known viruses present duplications, *Acanthamoeba polyphaga moumouvirus* (Yoosuf et al., 2012) and NYMV. This feature could have brought important adaptive advantages for both viruses, aiding the protection against deleterious mutations in the gene, allowing the emergence of novel mimivirus-like strains during evolutionary history and even potentially endowing the capacity of infecting a larger host range. For example, in the case of the methionyl-tRNA synthetase, duplications of the gene could have brought an important evolutionary advantage since its cognate amino acid is essential for the initiation of protein synthesis. For cysteinyl-tRNA synthetase, a high level of conservation is observed among the mimiviruses in which the genomes have already been described. This could have been important in facilitating the occurrence of an event of genetic duplication. Finally, the gene duplication of tyrosyl-tRNA synthetase could represent an important advantage for NYMV in the environment since its cognate amino acid ranks highly in the composition of the amino acid usage profile of mimiviruses (Silva et al., 2015). Beyond that, it was demonstrated in another study that apart from the conserved domains presented by aaRSs, which are involved in the process of aminoacylation, these enzymes might also incorporate novel motifs related to new biological functions in different eukaryotic organisms, for example functions related to angiogenic activity, angiostatic activity, and inflammatory response (Guo et al., 2010). Considering this case, the presence of duplicated aaRSs may represent a potential role for the addition of important new biological functions in giant viruses.

Another interesting fact was that from each duplicated copy of aaRS present in NYMV, one gene showed 100% identity with the corresponding APMV and SMBV gene, and the second copy also presented a 100% identity with a giant virus called KROV, isolated by our group from a different urban lake. This result may suggest an event of gene transfer among the ancestors of these viruses (Kroon vs. NYMV and or APMV-like virus and NYMV) in the same host at some moment in their evolutionary history. Furthermore, analysis of the expression of aaRS genes in NYMV showed that methionyl (duplicated) and tyrosyl-RS (duplicated) mRNA expression was significantly higher in cells infected with NYMV in comparison with APMV (**Figures 4A,B**), while for arginyl-RS (unduplicated) and cysteinyl-RS (duplicated), there was no significant difference in the expression (**Figures 4C,D**). Given the sequence similarity observed for the promoters of all of the aaRs genes (**Supplementary Figure S2**), we believe that this differential expression may be due to the presence of the duplicated genes in NYMV in comparison with APMV. This feature could give NYMV an advantage during its replication cycle into the host, increasing in the production of its own proteins during the process of translation and could be the cause of the faster growth of NYMV in comparison to APMV (**Figure 5**). The reason that cysteinyl RS NYMV duplication does not result in significant gene expression needs to be investigated, but it could be a result of some gene specificity regarding amoebal growth conditions (Silva et al., 2015) and/or virus host range.

The results obtained in this work suggest the importance of gene duplication events during the evolutionary history of the aaRSs in mimiviruses. These translation related genes seems to present a considerable influence during replication of the giant viruses in amoebae. This theme then becomes extremely interesting for future studies trying to understand the origin, genetic exchange and evolution of the aaRSs among mimiviruses, as well as the role of these processes in the acquisition of evolutionary advantages by the giant viruses.

## Author Contributions

PB, TA, LS, FA performed experiments and wrote the paper.

## Conflict of Interest Statement

The authors declare that the research was conducted in the absence of any commercial or financial relationships that could be construed as a potential conflict of interest.

## Footnotes

1 http://www.clcbio.com/index.php?id=28

2 http://enve-omics.ce.gatech.edu/aai/

## Supplementary Material

**FIGURE S1 | Comparative analysis of Niemeyer virus (NYMV) gene content with other mimivirus sequences**. The analysis showed the highest identity and bit-score distributions of NYMV against mimivirus group A sequences, such as Samba virus (SMBV) **(A)** and *Acanthamoeba polyphaga mimivirus* (APMV) **(B)**. Moreover, the similarity decreased toward moumouvirus **(C)** and Megavirus chilensis **(D)** of groups B and C, respectively. At the top of figures are mean and median of each compared group, considering identity and bit score distribution.

**FIGURE S2 | Promoter regions analysis of NYMV AaRS.** Red arrows indicate the promoter regions. Blue arrows indicate the start-codon regions.
