# Supplementary figures and images for: Niemeyer Virus: A New Mimivirus Group A Isolate Harboring a Set of Duplicated Aminoacyl-tRNA Synthetase Genes
Source: Front Microbiol. 2015 Nov 10;6:1256. doi: 10.3389/fmicb.2015.01256 (PMC4639698; doi:10.3389/fmicb.2015.01256)

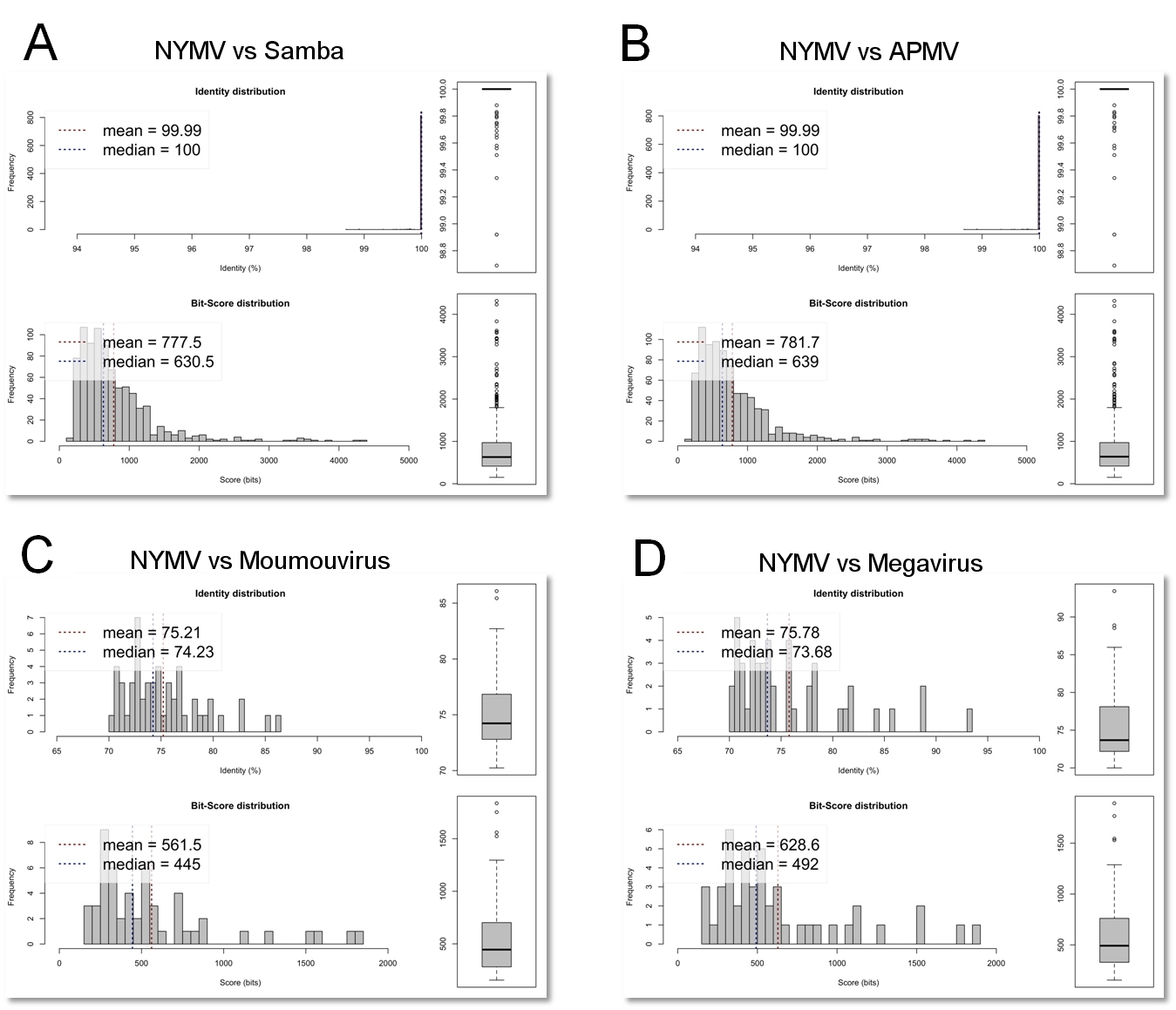

Supplement: Supplementary file 1 [file Image_1.JPEG]

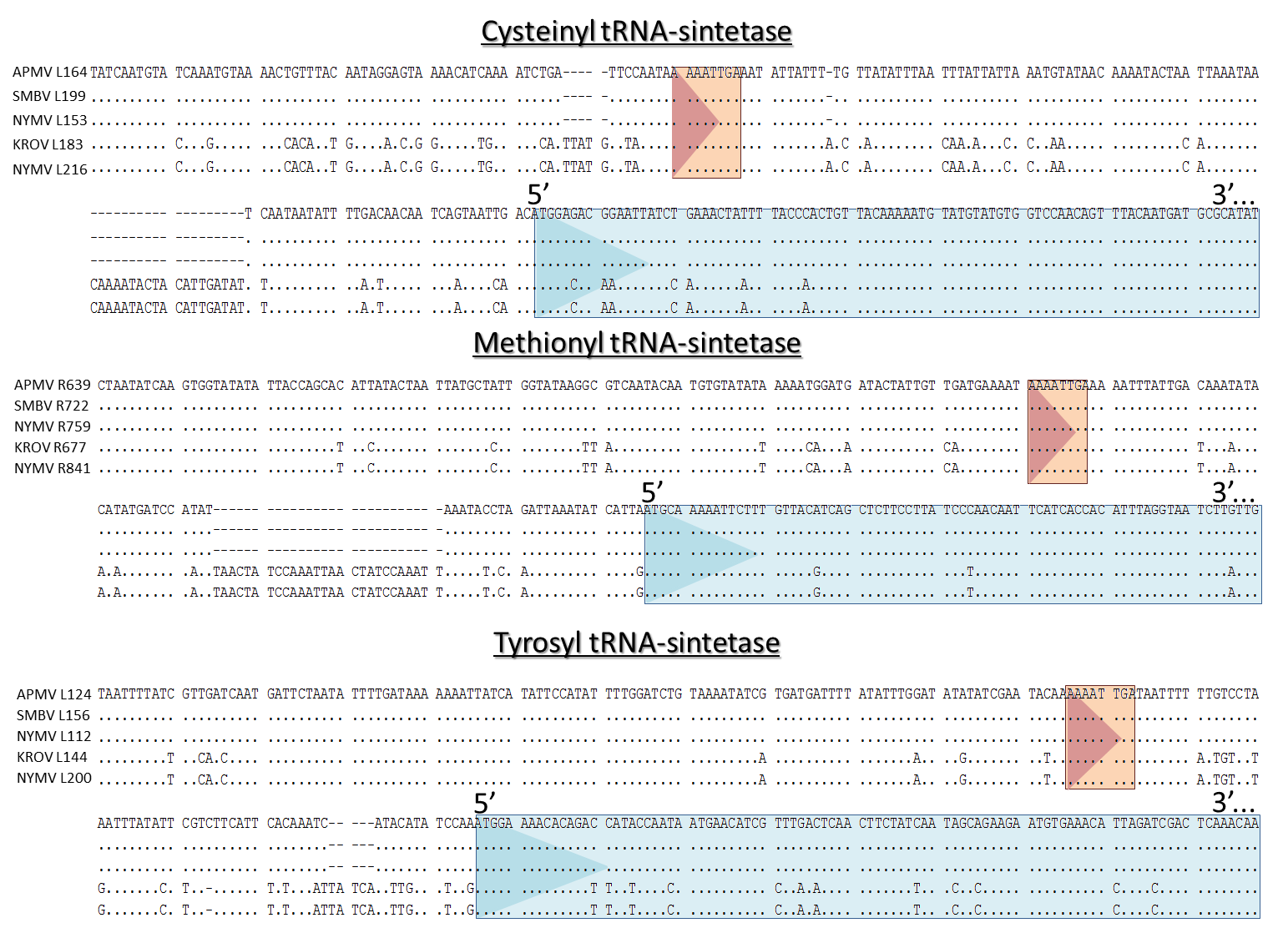

Supplement: Supplementary file 2 [file Image_2.TIF]
